# Ethanol extract of asiasari radix preferentially induces apoptosis in G361 human melanoma cells by differential regulation of p53

**DOI:** 10.1186/s12906-019-2609-2

**Published:** 2019-08-28

**Authors:** Kwang-Ha Park, Jeong-Hae Choi, Yeon-Suk Song, Gyoo-Cheon Kim, Jin-Woo Hong

**Affiliations:** 1 Department of Anatomy and Cell Biology, School of Dentistry, Yangsan Campus of Pusan National University, Beomeo-ri, Mulgeum-eup, Yangsan-si, Gyeongsangnam 50612 South Korea; 2 Department of Internal Medicine, School of Korean Medicine, Yangsan Campus of Pusan National University, Beomeo-ri, Mulgeum-eup, Yangsan-si, Gyeongsangnam 50612 South Korea

**Keywords:** Asiasari radix, Melanoma, p53, MDM2, CFLAR, Reactive oxygen species, Microarray

## Abstract

**Background:**

In Korea and China, asiasari radix (AR) is widely used as a traditional anti-inflammatory and analgesic agent. After its skin-regenerating and hair loss-preventing activities were identified, several types of AR extracts were used for aesthetic purposes. Nevertheless, the effect of ARE on various types of skin cancers was not fully studied yet.

**Methods:**

In this study, we tested the effect of an ethanolic AR extract (ARE) on G361 human melanoma and HaCaT human keratinocyte cell lines. After ARE exposure, cell growth and the expression patterns of proteins and genes were monitored.

**Results:**

The ARE-mediated cell growth inhibition was greater in G361 cells than in HaCaT cells due to differences in its cell growth regulation effects. Interestingly, ARE treatment induced caspase-3-mediated apoptosis in G361 cells, but not in HaCaT cells. Furthermore, ARE reduced the expression of p53 and p21 proteins in G361 cells, whereas it induced their expression in HaCaT cells. ARE induced cell death in G361 cells through the reactive oxygen species (ROS)-dependent regulation of p53 and p21 in G361 cells. Microarray analysis showed that ARE regulates Mouse double minute 2 homolog (MDM2) and CASP8 and FADD-like apoptosis regulator (CFLAR) gene expression in G361 and HaCaT cells differently.

**Conclusion:**

The treatment of ARE preferentially induces apoptosis in melanoma cells by the ROS-dependent differential regulation of p53 level. Therefore, ARE can be used as a new medicinal option for melanoma.

## Background

Asiasari radix (AR) has long been used in traditional Korean and Chinese medicine to treat cough, toothache, headache, neuralgia, gingivitis, asthma, and allergies due to its anti-bacterial and analgesic effects [1]. Recently, the pharmacological roles of AR extracts (AREs) have been reported. The anti-allergic [2, 3] and anti-inflammatory [4, 5] activities of AREs have been confirmed in vitro and in vivo. AREs also exert an anti-carries activity by not only reducing acid production, but also by inhibiting the growth and adhesion of *Streptococcus mutans* [6]. In addition, AREs also possess skin renewal and hair follicle-generating activities [7]. Furthermore, Jang et al. reported the possible skin-whitening role of ARE because it attenuates melanogenesis in rats [8]. Due to its potent skin regeneration and hair loss-preventing activities, AREs have been widely used in many cosmetics. Nevertheless, the effects of ARE on various types of skin cancers were studied poorly.

Melanoma is a type of skin cancer that accounts for about 4% of all cancers; however, it is the most dangerous since it accounts for about 80% of skin cancer-related deaths [9]. Although genetic risk factors contribute maximally to the development of melanoma, exposure to UV rays from the sun is directly or indirectly involved in the development of melanoma in 86% of the cases [10]. Fortunately, overall survival rate for patients with melanoma has gradually improved over the last 35 years due to improvement in detection systems along with surgical strategies. However, due to the lack of active agents for the treatment of melanoma, prognosis in patients diagnosed with malignant melanoma (stage IV) has remained grave [11]. One of the major goals of anti-cancer drug development is to selectively target cancer cells with high specificity [12]. Although numerous anti-melanoma drugs have been identified, the need for cancer cell-selective drugs is increasing gradually.

In this study, G361 human melanoma cells were treated with an ethanolic ARE for testing its role on cell proliferation and death. Furthermore, to compare the effects of ARE on keratinocytes with those on melanoma cells, we used HaCaT human keratinocytes to test whether ARE induces selective toxicity on melanoma cells. Furthermore, ARE-mediated changes in cell signaling pathways related with cell cycle regulation and apoptosis were determined using western blot analysis. In addition, the effects of ARE on gene expression patterns in the two cell lines were analyzed using cDNA microarray and RT-PCR analyses. Taken together, the results of this study indicate that ARE selectively induces apoptosis in melanoma cells, and presents an attractive approach for melanoma treatment.

## Methods

### Reagents

All chemicals were purchased from Sigma-Aldrich, Korea unless otherwise indicated.

#### Cell culture

HaCaT (which were used in our previous reports [13–15]) and G361 cells (purchased from ATCC®, Manassas,USA) were maintained in Dulbecco’s Modified Eagle’s Medium (DMEM, Gibco, Grand Island, NY, USA) supplemented with 1% penicillin/streptomycin (Gibco) and 10% fetal bovine serum (FBS, Gibco). Cells were incubated at 37 °C and 5% CO_2_.

#### ARE preparation

The root of *Asiasarum heterotropoides* (AR) was purchased from Hwalim pharmaceutical company (Seoul, South Korea). Dried AR (200 g) was finely ground and immersed in 2 l of 70% (v/v) ethanol at 60 °C for 16 h. The extracts were filtered, and excess solvent was evaporated under reduced pressure using a rotary evaporator at 40 °C. The powdered extract (21 g) was homogenized using a mortar and pestle, and stored at − 70 °C until further analysis. The recovery yield of the extracts was approximately 10% (w/w). A working solution of ARE was prepared by dissolving the powder in dimethyl sulfoxide (DMSO) that was further diluted to obtain suitable concentrations.

#### Cell growth assay using sulforhodamine B (SRB)

G361 and HaCaT cells were seeded in 24-well plates (1 × 10^5^ cells/well) and incubated for 24 h. For testing the dose-dependent effects of ARE, the cells were treated with 0, 200, 400, 600, 800, and 1000 μg/ml of ARE, and incubated for 24 h further. For testing the long-term effects of ARE, the cells were incubated for 24, 48, and 72 h in a growth medium containing 0, 200, 400, and 600 μg/ml of ARE. After incubation, the medium was gently removed, and the cells were fixed with 500 μl of 4% paraformaldehyde for 30 min at room temperature. After removing the paraformaldehyde, the cells were washed with tap water several times and stained with 0.4% SRB solution following the standard protocol of the SRB assay. After removing the SRB solution, the plates were washed with 1% acetic acid to ensure the complete removal of the SRB dye, and the plates were dried completely. The cells were imaged using optical microscopy, and 10 mM Tris buffer was added to each well to dissolve the SRB dye. SRB intensity in each sample was detected using UV spectroscopy at 515 nm.

#### SDS-PAGE and western blot analysis

G361 and HaCaT cells were seeded in 60-mm cell culture dishes (1 × 10^6^ cells). For testing the dose-dependent effects of ARE, the cells were treated with ARE at 200, 400, 600, 800, and 1000 μg/ml and incubated for 24 h. To evaluate the time-dependent effects of ARE, the cells were treated with 1 mg/ml of ARE at 2, 4, 6, 8, and 12 h. For the experiments using kinase inhibitors and NAC (N-acetyl-L-cysteine), the cells were exposed to the inhibitor 1 h before treatment with 1 mg/ml of ARE. The total incubation time of the cells was maintained as 48 h after cell seeding. After a final incubation, the cells were washed with PBS and harvested in ice-cold lysis buffer containing 50 mM Tris/HCl (pH 7.5), 150 mM NaCl, 1%(v/v) Nonidet P40, 10% (v/v) glycerol, 1 mM PMSF, 1 mM dithiothreitol, 20 mM NaF, 1 mM EDTA, and a protease inhibitor cocktail (Roche). Equal amounts of cell lysate (30 μg) were resolved by SDS/PAGE (8–12% gel) and transferred to PVDF membranes. Upon the completion of transfer, the membranes were probed with antibodies against poly (ADP-ribose) polymerases (PARP), p53, p21, c-Jun N-terminal kinases (JNK), phospho-JNK, p38α/β, phospho-p38, cyclin A, cyclin D1 (Santa Cruz Biotechnology, CA, USA), and cleaved-caspase-3 (Cell Signaling Technology, MA, USA). The specific bands were detected with advanced ECL western blotting reagents (Merck Millipore, Darmstadt, Germany). An anti-GAPDH antibody was used as a loading control (Santa Cruz Biotechnology).

#### cDNA microarray

G361 cells and HaCaT cells were seeded in 60-mm cell culture dishes for 48 h. Further, the cells were treated with 1 mg/ml of ARE and incubated for 4 h. Cellular RNA was isolated using the TRIzol™ reagent (Thermo Fisher Scientific, DE, USA). The extracted RNA purity was determined at 260/280 nm using a NanoDrop 1000 spectrophotometer (Thermo Fisher Scientific) before using the RNA samples for cDNA microarray analysis (GenomicWorks, Daejeon, Korea).

#### RT-PCR analysis

G361 cells and HaCaT cells were seeded in 60-mm cell culture dishes and incubated for 48 h. Further, the cells were treated with 1 mg/ml of ARE for 4 h, and total RNA was isolated using the TRIzol™ reagent (Thermo Fisher Scientific). The specific methods for RT-PCR can be found in our previous report [13]. Specific primers against *MDM2* (sense: CGGAACAAGAGACCCTGG, antisense: GAGTCCGATGATTCCTGC TG) and *CFLAR* (sense: TGCCCTTATCTAGCAGGGA, antisense: CAGGAGTGGGCGTTTTCT) were used. After PCR analysis, the samples were electrophoresed on 1.5% agarose gel containing ethidium bromide. The gels were imaged by ImageQuant Las (GE Healthcare Life Sciences, Freiburg, Germany) image analyzer.

#### Data analysis

Data are presented as the mean ± standard error of the mean (SEM) of at least three independent experiments. The two-tailed Student’s *t*-test was used to assess statistical significance for differences in means, and the significance was set at *p* < 0.05.

## Results

### The treatment of ARE preferentially inhibits the growth of G361 human melanoma cells in a dose-dependent manner

**Fig. 1:**
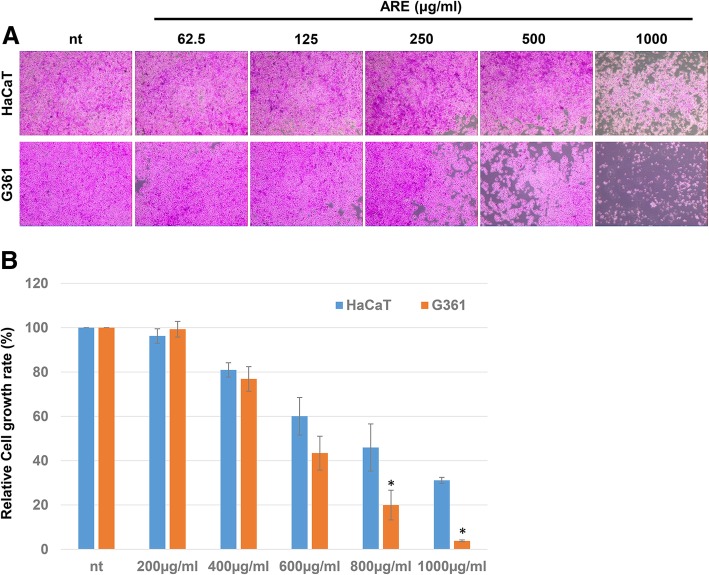
Dose-dependent effects of ethanolic extract of asiasari radix (ARE) on the growth of G361 melanoma cells and HaCaT keratinocytes. **a** Microscopic images of G361 and HaCaT cells after 24 h of incubation with 0, 62.5, 125, 250, 500, and 1000 μg/ml of ARE. Total cellular protein was stained with SRB; data shown are representative of three independent experiments. **b** Dose-dependent effects of ARE (200, 400, 600, 800 and 1000 μg/ml) on the growth rate of G361 and HaCaT cells were tested at 24 h after the treatment, using the SRB assay. Data represent the mean ± SEM (*n* = 4). The significant difference between HaCaT and G361 cells were marked as * (*p* < 0.05); nt, non-treated control

The effects of ARE on the growth of G361 melanoma cells and HaCaT human normal keratinocytes were tested. At first, the cells were treated with ARE at concentration of 62.5, 125, 250, 500 and 1000 μg/ml for 24 h and then the cell density were monitored. The results showed that ARE treatment significantly decreased the growth of G361 cells in a dose-dependent manner (Fig. 1a). Interestingly, although 1 mg/ml of ARE also decreased the cell density of HaCaT cells, the decrease was lesser in HaCaT cells than in G361 cells. This phenomenon was confirmed further by monitoring the effect of ARE (at concentration of 200, 400, 600, 800 and 1000 μg/ml) on the growth rates of the two cells at 24 h after the treatment (Fig. 1b). ARE at concentrations above 600 μg/ml showed higher growth inhibition in G361 cells than in HaCaT cells. Especially, 37% of HaCaT cells were still remained at 24 h after the treatment with 1 mg/ml of ARE; however, only 3% of G361 cells were survived at same condition.

**Fig. 2:**
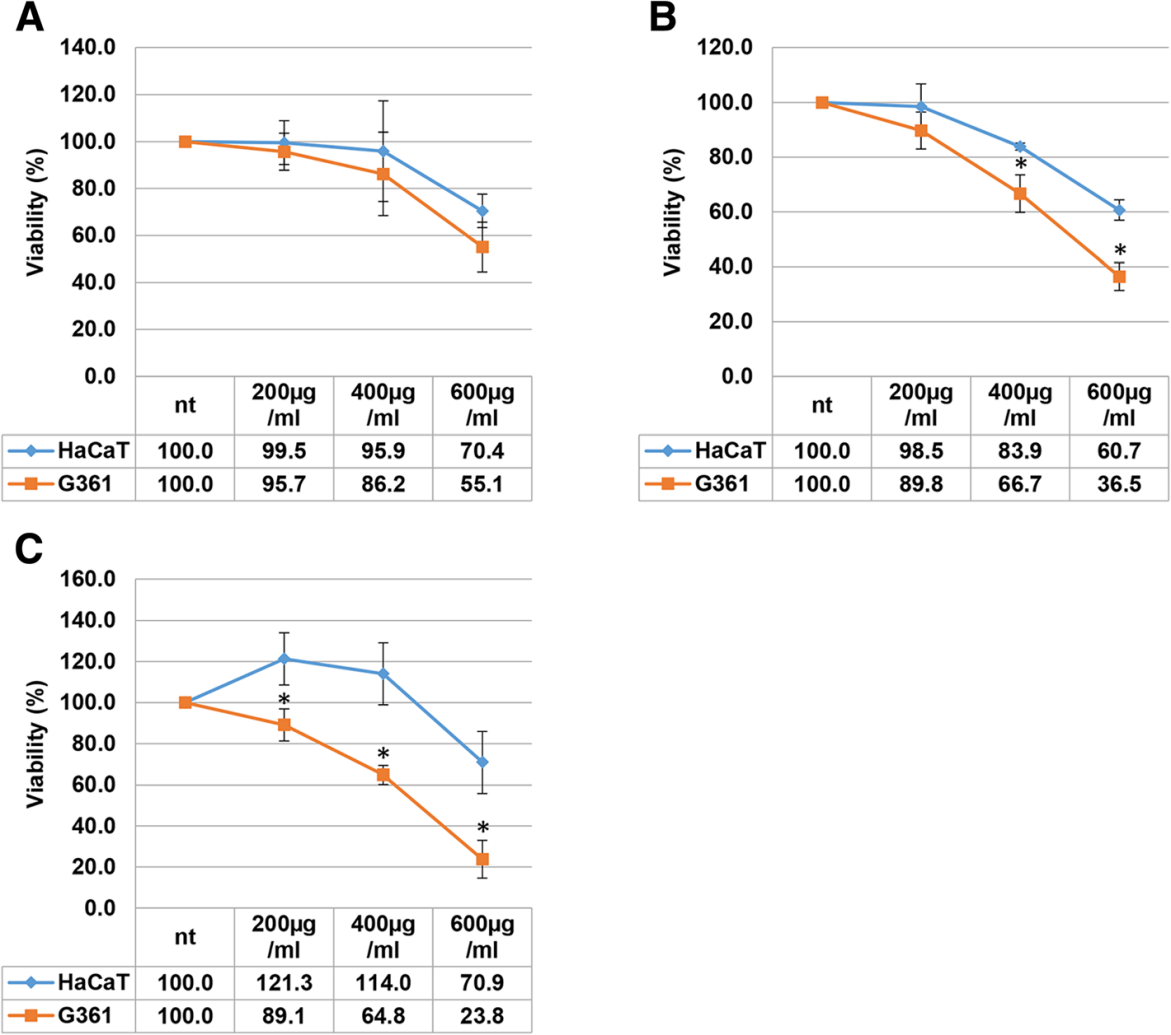
Long-term effects of ARE on the growth of G361 and HaCaT cells. The growth of G361 and HaCaT cells were monitored at 24 (**a**), 48 (**b**), and 72 h (**c**) after ARE treatment at concentrations of 200, 400, and 600 μg/ml using the SRB growth assay. Data represent the mean ± SEM (*n* = 4). The significant difference between HaCaT and G361 cells were marked as * (*p* < 0.05)

Further, the cells were treated with ARE at 200, 400, and 600 μg/ml, and the growth of G361 and HaCaT cells were monitored at 24, 48, and 72 h after treatment. Similar to our previous results, the effects of ARE did not show significant differences between the two cell lines at all concentrations at 24 h after treatment (Fig. 2a). However, at 48 h after treatment, the growth inhibition effects of ARE on G361 cells were much greater than those on HaCaT cells, and significant differences between the two cells were observed at concentrations of 400 μg/ml or greater (Fig. 2b). Interestingly, ARE treatment at 200 and 400 μg/ml for 72 h slightly increased the growth of HaCaT cells; however, it decreased the growth of G361 melanoma cells (Fig. 2c).

### The treatment of ARE induces apoptosis in G361 cells but not in HaCaT cells

**Fig. 3:**
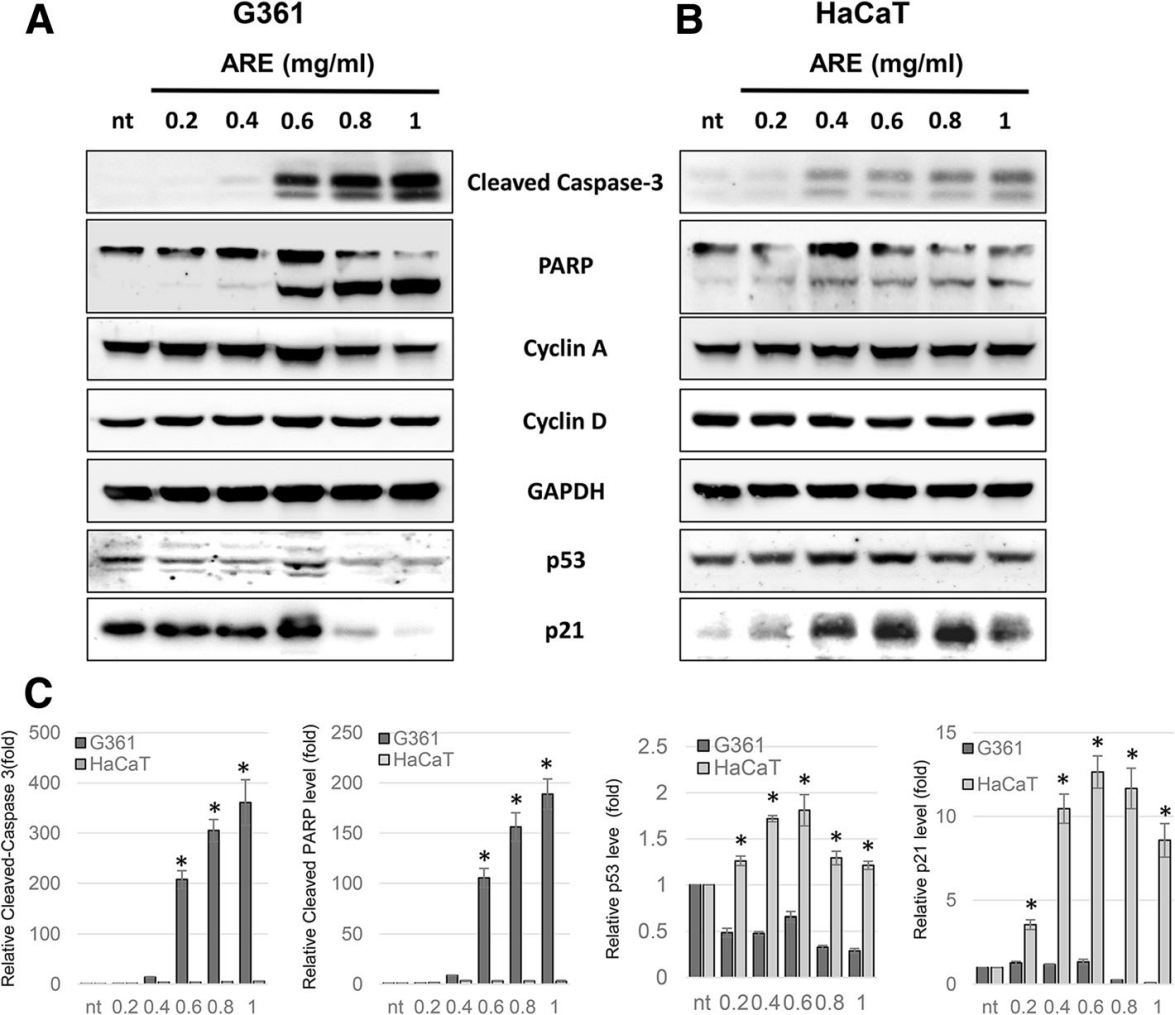
Dose-dependent effects of ARE on the expression of apoptosis- and cell cycle-related proteins in G361 and HaCaT cells. The results of western blot analysis in G361 (**a**) and HaCaT (**b**) cells treated with 0.2, 0.4, 0.6, 0.8, and 1 mg/ml of ARE for 24 h are shown. GAPDH was used as a loading control. **c** The relative density of each protein of (A) and (B). Data represent the mean ± SEM (*n* = 3). The significant difference between HaCaT and G361 cells were marked as * (*p* < 0.05)

For a more detailed understanding of the differential effects of ARE on G361 and HaCaT cells, the expression of molecular markers of cell death and growth arrest were monitored (Fig. 3). The treatment of ARE greatly increased the expression of apoptotic cell death marker proteins, cleaved caspase-3 and PARP, in G361 cells in a dose-dependent manner (Fig. 3a) but not in HaCaT cells (Fig. 3b). The effect of ARE on cell cycle-regulating cyclins were not significant in both cells. However, ARE-mediated changes in the expression of p21 and p53 proteins in G361 cells were significantly different from those in HaCaT cells. The treatment of ARE decreased the expression of p53 in G361 cells but not in HaCaT cells. Furthermore, the expression of p21 in G361 cells was significantly reduced by ARE treatment in a dose-dependent manner; however, ARE significantly increased the expression of p21 in HaCaT cells.

#### The treatment of ARE induces the rapid cleavage of caspase-3 and PARP in G361 cells by decreasing p21 and p53 expression

**Fig. 4:**
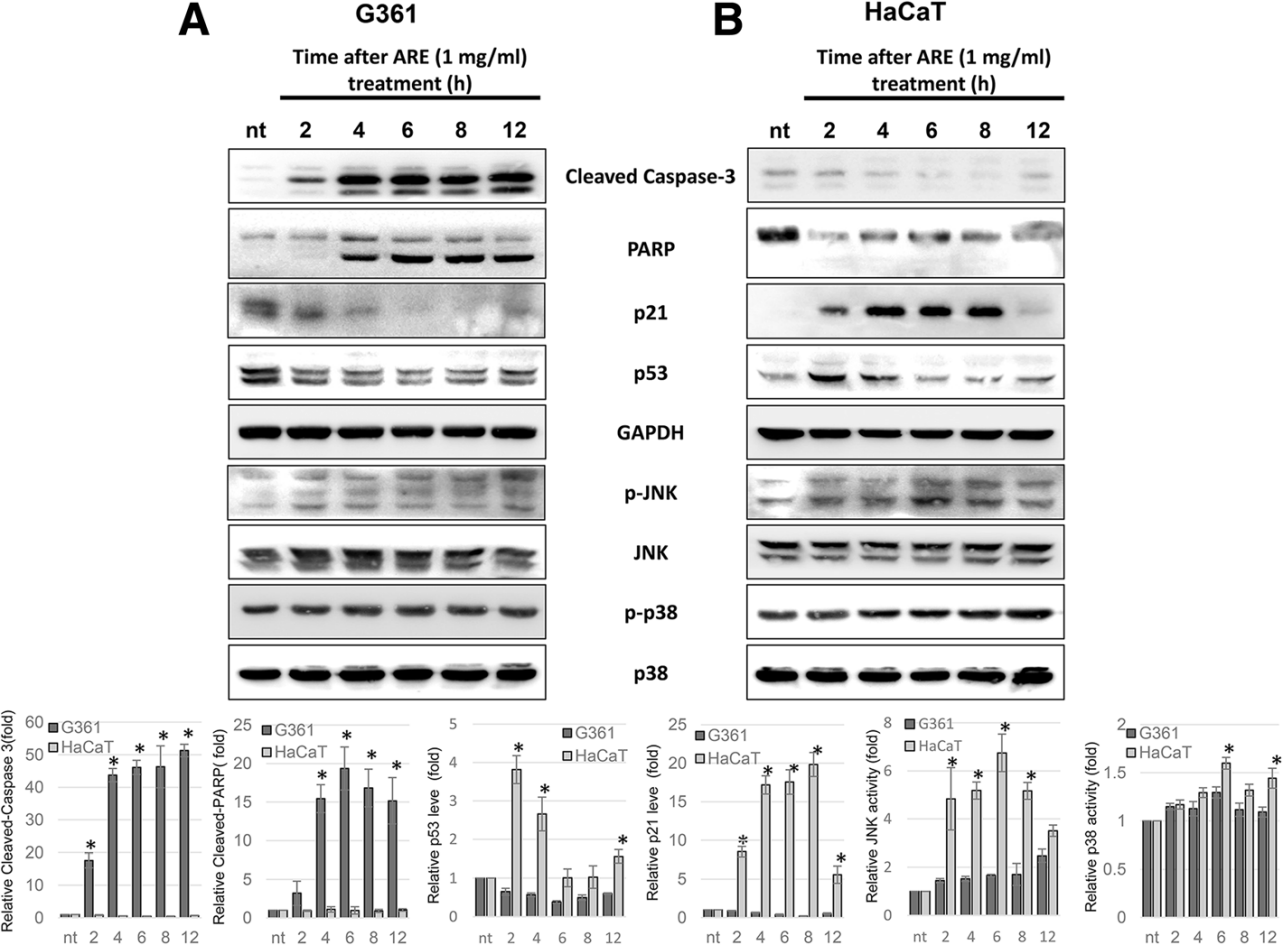
Time-dependent effects of ARE on the expression of apoptosis- and stress-related proteins in G361 and HaCaT cells. The results of western blot analysis in G361 (**a**) and HaCaT (**b**) cells treated with 1 mg/ml of ARE for 2, 4, 6, 8, and 12 h are shown. The relative density of each proteins of (**a**) and (**b**) were represented as graphs (bottom panel). Data represent the mean ± SEM (*n* = 3). The significant difference between HaCaT and G361 cells were marked as * (*p* < 0.05)

To identify cellular stress-related signaling proteins responsible for ARE-mediated apoptosis, the effect of ARE on the activities of JNK, p38, and other apoptosis-related proteins in G361 and HaCaT cells were monitored at several time points after treatment. ARE at 1 mg/ml immediately induced the apoptosis in G361 cells by increasing the cleavage of caspase-3, and cleaved PARP was detected at 4 h after treatment (Fig. 4a). Furthermore, ARE-mediated decrease in p21 and p53 was detected at 2 h after treatment. However, ARE treatment did not induce the apoptotic cleavage of caspase-3 and PARP in HaCaT cells (Fig. 4b). In fact, it significantly increased the expression of p21 and p53 proteins in HaCaT cells. In both cells, ARE slightly increased the phosphorylation of JNK; however, p38 activation was observed only in HaCaT cells.

### The ARE-mediated apoptosis in G361 cells is mediated by reactive oxygen species (ROS)-dependent decrease in p21 and p53

**Fig. 5:**
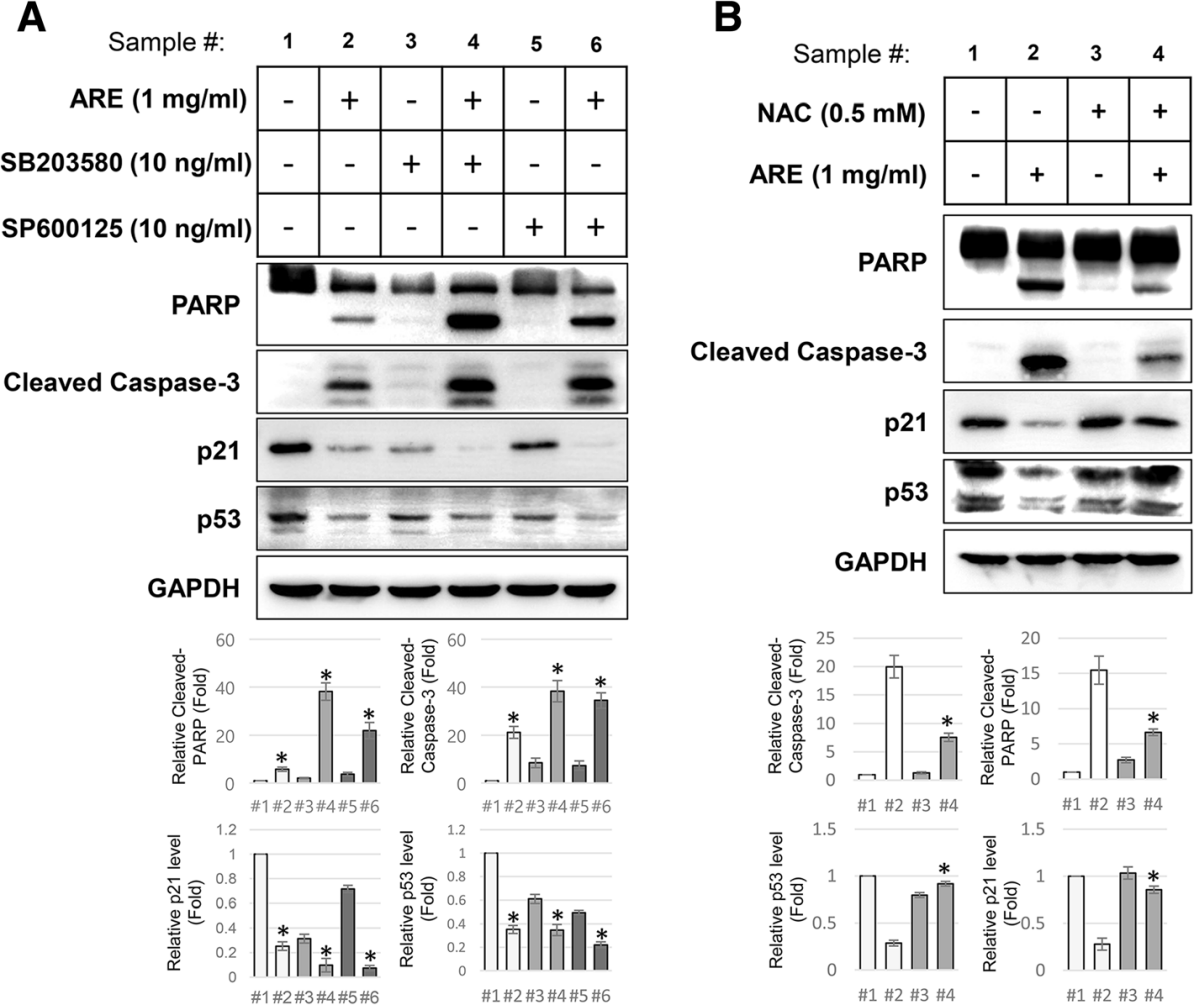
ROS-dependent ARE-mediated apoptosis and the expression of p53 and p21 proteins in G361 cells. **a** The effects of specific inhibitors of JNK (SP600125, 10 ng/ml) and p38 (SB203580, 10 ng/ml) on ARE-mediated apoptosis and the expression of p53 and p21 proteins were tested using western blotting. The cells were pretreated with the inhibitors for 1 h before ARE (1 mg/ml, 4 h) treatment. The relative density of cleaved caspase-3 and –PARP, along with p21 and p53 were represented as graphs (bottom panel). Data represent the mean ± SEM (n = 3). The significant changes after ARE treatment were marked as * (*p* < 0.05) (**b**) The results of western blot analysis showing the effects of an ROS scavenger (NAC, 0.5 mM) on ARE-mediated effects in G361 cells are shown. The cells were pretreated with NAC for 1 h before ARE treatment. The relative density of each proteins were represented as graphs (bottom panel). Data represent the mean ± SEM (*n* = 3). The significant changes after ARE treatment were marked as * (*p* < 0.05)

The roles of JNK and p38 proteins in ARE-mediated apoptosis in G361 cells were identified using specific kinase inhibitors. Pretreatment with SB203580, a specific inhibitor of p38, decreased the basal expression of p21 and p53 proteins, and failed to block ARE-mediated decrease in the levels of these proteins (Fig. 5a). Furthermore, the inhibition of p38 enhanced ARE-mediated apoptosis in G361 cells. The effects of JNK inhibition (using SP600125) were similar to those of p38 inhibition; however, its effect on the ARE-mediated cleavage of PARP was milder than that of p38 inhibition.

The treatment of ARE on various types of cancer cells induces intracellular ROS production. To determine whether ROS-mediated signaling stimulates ARE-mediated apoptosis in G361 cells, the effect of a pan-ROS inhibitor, NAC, on ARE-mediated changes in G361 cells was tested. NAC not only reduced the ARE-mediated cleavage of caspase-3 and PARP, but also blocked ARE-mediated decrease in p21 and p53 protein levels (Fig. 5b).

### The treatment of ARE differentially regulates ROS- and apoptosis-related gene expression in G361 and HaCaT cells

To elucidate the mechanism of the ARE-mediated regulation of p21 and p53 expression and apoptosis in G361 and HaCaT cells, the cells were treated with 1 mg/mL of ARE, incubated for 4 h, and the total cellular RNA extracts were used for cDNA microarray analysis. The treatment of ARE altered the expression of 4359 out of 58,285 probes (increase in the expression of 2650 probes and decrease in the expression of 1709 probes) by at least 2-fold in G361 cells. However, it induced a 2-fold change in the expression of only 2304 probes (increase in the expression of 1286 probes and decrease in the expression of 1018 probes) in HaCaT cells.

**Fig. 6:**
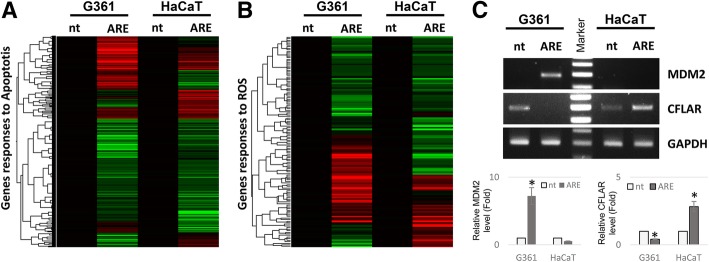
cDNA microarray-based identification of *MDM2* and *CFLAR* as genes responsible for ARE-mediated apoptosis in G361 cells. Genes showing more than 2-fold change after ARE treatment identified by cDNA microarray analysis using total RNA from G361 and HaCaT cells were classified into two groups; (**a**) apoptosis-related genes and (**b**) ROS-related genes. The change in gene expression levels in each group after ARE treatment is expressed as a heat map. The color intensity represents the extent of change; green indicates a decrease in gene expression, whereas red indicates an increase in gene expression. **c** The results of RT-PCR analysis of *MDM2* and *CFLAR* genes are shown. GAPDH was used as a loading control. The relative expression of MDM2 and CFLAR mRNA were represented as graphs (bottom panel). Data represent the mean ± SEM (*n* = 3). The significant changes after ARE treatment were marked as * (*p* < 0.05) Table 1. The list of ROS and apoptosis related genes those differently regulated by ARE in G361 and HaCaT cells. The significant changes after ARE treatment were marked as * (*p* < 0.05)

To narrow down candidate genes responsible for the ARE-mediated differential regulation of p21 and p53 that induce apoptosis in G361 and HaCaT cells, all probes related with apoptosis were searched under a standard list of genes (www.geneontology.org) using ‘regulation of apoptotic process’ as the keyword; ARE-mediated changes (1065 probes) in both cells are shown as a heat map (Fig. 6a). Because ARE-mediated cell death was effectively blocked by an ROS inhibitor, we searched for genes related to ROS production using ‘cellular response to ROS’ as the keyword; ARE-mediated changes (133 probes) are expressed as a heat map (Fig. 6b). Although some probes showed similar changes in both cells, others (109 probes in Fig. 6a and 18 probes in Fig. 6b) showed contrasting ARE-mediated changes in the two cell lines.

To further narrow down candidate genes responsible for the ARE-mediated preferential regulation of apoptosis in HaCaT and G361 cells, apoptosis- and ROS-related probes (Fig. 6a and b) that were oppositely affected by ARE treatment in the two cell lines were selected. These probes are described in Table 1. Among five key candidate genes, we focused on the differential effects of ARE on *MDM2* and *CFLAR* (CASP8 and FADD-like apoptosis regulator) in G361 and HaCaT cells. The expression of *MDM2* (a negative regulator of p53) was upregulated (2.89-fold) by ARE in G361 cells; however, it was downregulated (approximately 2-fold) in HaCaT cells. The other gene, CFLAR, also known as c-FLIP (FLICE-like inhibitory protein), is well known for its anti-apoptotic activity and is positively regulated by the p53 protein. The results of cDNA microarray analysis showed that ARE reduces (2.85-fold) *CFLAR* expression in G361 cells; however, it induces (2-fold) *CFLAR* expression in HaCaT cells. These results were further confirmed by performing RT-PCR analysis (Fig. 6c).

**Table 1:** ROS and apoptosis related genes those differently regulated by ARE in G361 and HaCaT cells

Gene Name	Gene Symbol	UniGene ID No.	GeneBank No.	Locus	Mean Fold G361 (*p* value)	Mean Fold HaCaT (*p* value)
PRKC, apoptosis, WT1,regulator	PAWR	Hs.643130	NM_002583	12q21.2	3.10* (0.042)	0.94 (0.352)
MDM2, proto-oncogene, E3 ubiquitin protein ligase	MDM2	Hs.484551	NM_002392	12q15	2.89* (0.005)	0.50* (0.038)
Histone deactylase 2	HDAC2	Hs.3352	NM_001527	6q21	5.32* (0.035)	0.37* (0.048)
Receptor (TNFRSF)-interesting serine-threonine kinase 1	RIPK1	Hs.519842	NM_003804	6p25.2	0.32* (0.018)	1.07 (0.418)
CASP8 and FADD-like apoptosis regulator	CFLAR	Hs.390736	NM_001202519	2q33.1	0.35* (0.001)	2.00* (0.029)

The significant changes after ARE treatment were marked as * (*p* < 0.05)

## Discussion

Studies on the effects of AREs on the skin and hair have mainly focused on aesthetical benefits, including skin regeneration, skin whitening, and the prevention of hair loss. Therefore, AREs are commonly used in herbal skin-care products. However, their effects on various types of skin cancer cells have not been fully studied. Although they have been reported to exhibit anticancer effects on other types of cancers, including colon cancer [16], lung cancer [17, 18], and cervical cancer, specific mechanisms of action have not been uncovered.

Melanoma can be formed by malignant transformation of melanocytes, the cells in the skin responsible for pigment production [19]. Although the early stage melanoma can be easily removed surgically, the late stage malignant melanoma cannot be treated easily because of their lack of responsiveness to currently available therapies [20]. In this study, the possible anticancer effects of an ethanolic ARE was tested against malignant melanoma. For this, the most frequently used G361 human melanoma cell line, which is also capable of melanogenesis was used [21]. To identify whether its actions were selective to cancer cells, its effects on HaCaT human keratinocytes were evaluated. Although HaCaT is an artificially constructed immortalized cell line, these cells can differentiate into epidermal cells of the skin [22]. Although treatment with ARE for 24 h reduced the proliferation of both cells, its effects on G361 were more severe than those on HaCaT cells at ARE concentrations of 0.8 mg/ml and greater (Fig. 1). Although the effects of ARE at concentrations of 0.2–0.6 mg/ml on HaCaT and G361 cells were similar at 24 h after treatment, ARE preferentially downregulated the growth of G361 cells at 48 and 72 h after treatment (Fig. 2). However, ARE treatment at concentrations of 0.2 and 0.4 mg/ml for 72 h slightly enhanced the growth of HaCaT cells. This finding corroborates previous reports on hair growth-promoting effects of AREs based on the increased proliferation of HaCaT and dermal papilla cells after low-dose ARE treatment [7]. These results indicate that the long-term exposure of the skin to AREs can selectively reduce the growth of melanoma cells. Although Lee et al. have reported that AREs preferentially inhibit the growth of A549 lung cancer cells [17], the mechanism of its selective anticancer effect remains unexplored.

Therefore, the effects of the ethanolic ARE on the expression of apoptosis- and cell cycle-related proteins in G361 and HaCaT cells were determined. ARE effectively increased the cleavage of caspase-3 and PARP in G361 cells in a dose-dependent manner; however, no ARE-mediated change in these two proteins was detected in HaCaT cells (Fig. 3). Because the cleavage of these two proteins indicate apoptosis, it may be considered that the ARE-mediated growth inhibition of G361 cells is caused by apoptosis; however, ARE reduces the growth of HaCaT cells by other mechanisms. Although ARE treatment on the two cell lines did not modulate the expression of cell cycle-promoting proteins, the expression of p53 and p21, negative regulators of the cell cycle, were differently affected by ARE in G361 and HaCaT cells. In G361 cells, ARE treatment decreased the expression of p53 and p21 proteins, whereas in HaCaT cells, the expression of these two proteins was increased by ARE.

This differential effect of ARE on the cleavage of apoptotic proteins, and the expression of p21 and p53 in the two cell lines were confirmed in our time-dependent experiments (Fig. 4). In G361 cells, the cleavage of caspase-3 was induced at 2 h after ARE treatment, and PARP cleavage occurred after 4 h. However, ARE treatment on HaCaT cells failed to induce the cleavage of caspase-3 and PARP until 12 h after treatment. Caspase-3 activation occurs last in the apoptotic caspase activation cascade, and cleaved-caspase-3 actively promotes the cleavage of its substrate proteins, including PARP, leading to apoptosis [23]. PARP is normally overexpressed when cell DNA is damaged, and actively participates in DNA repair process. However, when DNA damage is too severe, PARP undergoes caspase-3-mediated cleavage to form an apoptosis-promoting protein [24]. Therefore, the effects of ARE on the cleavage of caspase-3 and PARP in G361 cells indicate the ARE-mediated activation of the apoptotic caspase cascade. Along with apoptotic protein regulation, the ethanolic ARE differently regulated the expression of p53 and p21 proteins in the two cell lines. In G361 cells, ARE treatment immediately reduced the expression of p53 and p21 protein; however, their expression was increased in HaCaT cells. p53 is a major tumor suppressor protein, which is reported to be mutated in several cancers including melanoma. As a multi-functional protein, p53 actively regulates the expression of DNA repair and apoptosis-promoting genes [25]. *p21* gene expression is positively regulated by p53. As a negative regulator of the cell cycle, p21 not only induces growth arrest, but also delays apoptosis under severe stress conditions [26]. Therefore, as shown in Fig. 4, ARE differentially regulates the expression of p53 and p21, and causes apoptosis in G361 cells and growth arrest in HaCaT cells.

What kind of stress-mediated signal pathway is participating in ARE-mediated p53 and p21 decrease and apoptotic cell death in G361 cells? According to the results shown in Fig. 5a, JNK and p38 kinases, well-known stress-signal mediators, are not responsible for ARE-mediated cell death in G361 cells. However, treatment with p38 and JNK inhibitors enhanced the ARE-mediated cleavage of caspase-3 and PARP, and decreased the expression of p53 and p21 proteins. These effects might be because of the multi-functional properties of p38 and JNK. Although JNK and p38 proteins induce apoptosis under several stress conditions, they can be activated by growth factors to promote anti-apoptotic gene regulation [27, 28]. Especially, the role of p38 in apoptosis regulation is dependent on the type of cells, and Yee et al. reported that it causes G1 phase cell cycle arrest by activating p21 [29]. Accordingly, we observed that the inhibition of p38 further led to the ARE-mediated decrease in p21 activation, thereby causing enhanced ARE-mediated apoptosis in G361 cells.

In many kinds of cancer cells, excessive ROS can promote apoptotic cell death [30], and several natural plant-derived compounds have been reported as potential anti-cancer drugs inducing ROS-dependent apoptosis [31]. In this study, ARE-mediated apoptosis in G361 cells was ROS-dependent. Treatment with NAC, an ROS scavenger, significantly reduced ARE-mediated caspase-3 and PARP cleavage, and apoptosis, and decrease in p53 and p21 protein expression was prevented. These data indicate that an increase in cellular ROS decreases p53 and p21 protein expression to cause ARE-mediated apoptosis in G361 cells.

Finally, candidate genes responsible for the ARE-mediated differential regulation of apoptosis, and p53 and p21 expression in G361 and HaCaT cells were identified by performing a cDNA microarray analysis. Because ARE-mediated apoptosis occurred only in G361 cells, we measured changes in the expression of apoptosis-related genes (Fig. 6a). Because ARE induced apoptosis in G361 cells in an ROS-dependent manner, changes in the expression of ROS-related genes were also studied (Fig. 6b). Some genes showed similar expression patterns after ARE treatment in both the cell types; however, other genes were modulated differently by ARE. Despite being exposed to the same stress stimulus, ARE differentially regulated p53 and p21 expression, and apoptosis in G361 and HaCaT cells. As shown in Table 1, five genes were identified to be related to apoptosis and ROS that were differentially regulated by ARE in G361 and HaCaT cells. Among them, the expression of *MDM2* and *CFLAR* genes is directly related with p53 protein level. As one of major negative regulators of p53, MDM2 protein promotes the ubiquitin-mediated degradation of p53 protein [32, 33]. On the other hand, *CFLAR* is one of the target genes of p53 protein, and it binds to the death receptor signaling complex and inhibits apoptosis by blocking the activation of caspases [34, 35]. As shown in Fig. 6c and Table 1, ARE induces *MDM2* gene expression in G361 cells, whereas it decreases its expression in HaCaT cells. The expression pattern of *CFLAR* gene after ARE treatment was similar to that of p53; its expression decreased in G361 cells, whereas it increased in HaCaT cells. Therefore, these results indicate that ARE-mediated increase in ROS induces the expression of *MDM2* in G361 cells, which can be directly linked to the decrease in p53 protein level and its target gene *CFLAR*. Because CFLAR is a caspase inhibitor, caspase-mediated apoptosis in G361 cells upon ARE treatment is plausible. However, ARE reduced *MDM2* gene expression and stimulated p53 protein and *CFLAR* gene expression in HaCaT cells, leading to the blockage of ARE-mediated caspase activation in these cells. In the future, we propose to elucidate the mechanism of the differential regulation of *MDM2* gene expression by ARE in the two types of cells.

## Conclusion

In this study, the anticancer activity of ARE, which is frequently exposed to the skin as a component of aesthetic products was tested using a melanoma cell line The results of this study elucidate the noble anti-melanoma activity of ARE, which preferentially induces apoptosis in G361 melanoma cells rather than in immortalized keratinocytes. In addition, specific mechanisms for its anticancer activity have been suggested. ARE increases cellular ROS and differentially regulates the expression of *MDM2* gene in melanoma cells, thereby decreasing the expression of anti-apoptotic genes, such as *p21* and *CFLAR*, by negatively regulating p53 protein expression. Taken together, this study presents the possible roles of ARE as an anti-melanoma agent apart from its effects on skin regeneration and hair loss prevention.

## Data Availability

All data generated or analyzed during this study are included in this published article.

## Abbreviations

AR: Asiasari radix
ARE: Ethanol extract of AR
CFLAR: CASP8 and FADD-like apoptosis regulator
MDM2: Mouse double minute 2 homolog
NAC: N-acetyl-L-cysteine
PARP: Poly (ADP-ribose) polymerases
ROS: Reactive oxygen species
SRB: Sulforhodamine B
